# Editorial: Coordination of plant endomembrane system with developmental signals and environmental stimuli

**DOI:** 10.3389/fpls.2022.977333

**Published:** 2022-08-02

**Authors:** Ruixi Li, Georgia Drakakaki, Yansong Miao, Ying Fu, Xiaojuan Li

**Affiliations:** ^1^Key Laboratory of Molecular Design for Plant Cell Factory of Guangdong Higher Education Institutes, Institute of Plant and Food Science, Department of Biology, School of Life Sciences, Southern University of Science and Technology, Shenzhen, China; ^2^Department of Plant Sciences, University of California, Davis, Davis, CA, United States; ^3^School of Biological Sciences, Nanyang Technological University, Singapore, Singapore; ^4^State Key Laboratory of Plant Physiology and Biochemistry, Department of Plant Sciences, College of Biological Sciences, China Agricultural University, Beijing, China; ^5^Key Laboratory of Genetics and Breeding in Forest Trees and Ornamental Plants Ministry of Education, College of Biological Sciences and Biotechnology, Beijing Forestry University, Beijing, China

**Keywords:** plant endomembrane system, biotic stress, abiotic stress, cytoskeleton dynamics, intracellular transport

Being sessile, plants have developed many plant-specific mechanisms to adapt to their unique lifestyle and respond to the changing environment. For a long time, researchers in plant biology have focused on the hormone regulation pathways and signal transduction cascades, thus the roles of endomembrane proteins in developmental program and stress response remained largely unexplored. During the past decade, however, researchers have started to unravel the mysteries of the endomembrane system under physiological conditions by integrating image analysis with forward and reverse genetics approach. Recent progresses have shed light on the extensive interactions between endomembrane proteins and signaling pathways.

In this Research Topic collection, we published nine papers, which could be grouped into three categories. In the first category, researchers reviewed recent advances about special organelles or trafficking modulators in developmental regulation and stress response. Liu, Kang et al. summarized the current understanding of plant extracellular vesicles (EVs), including their isolation technologies, biogenesis and essential roles in plant immunity. EVs are potential carriers for metabolites and nucleotides with key roles in cross-species regulation, therefore this paper has drawn broad attention after publication. Mao and Tan reviewed recent progress in the biological functions of SUPPRESSOR OF ACTIN (SAC) domain-containing phosphoinositide phosphatases in plants. Considering the vital role of phosphatidylinositol in a wide range of biological processes, this mini review provides novel insights into its regulatory mechanism. Li et al. discussed recent advances in the organization and dynamics of actin filaments and microtubule networks in guard cells, emphasizing cytoskeletal rearrangements during stomatal movement. Stomata are the main sites for gas exchange in leaves, so their regulatory mechanisms are important to ensure efficient photosynthesis. Law et al. highlighted recent findings and potential applications of adaptor protein (AP) complexes, retromer, and retriever complexes in post-Golgi trafficking.

In the second category, the authors focused on studying a particular endomembrane protein under different physiological conditions. Xu et al. described the essential function of the aquaporin gene MaPIP1;1 in regulating multiple abiotic stresses in banana. Wang et al. showed that the constitutive active calcium-dependent protein kinase 30 (CPK30) plays an important role in root growth regulation and endomembrane trafficking. Sun et al. demonstrated that Root Hair Defective3 (RHD3) acts as an ER-phagy receptor under ER stress to promote ER-phagy in *Arabidopsis*. RHD3 is the close homolog of the Atlastin-type GTPase in mammalian cells, which has been widely reported as the key regulator of ER fusion and ER-phagy. This is the first paper in plant field revealing the conserved role of RHD3 as an important ER-phagy receptor in *Arabidopsis*.

In the third category, the researchers discussed cell biological aspects in plant species other than model plant *Arabidopsis*. Liu, Shen et al. reviewed the current knowledge about vacuoles in Bryophytes, including their special properties, biogenesis mechanism and evolutionary roles. Liu, Zhang et al. tested different GFP-ATG8 markers to monitor autophagy in rice. Since both vacuolar transport and autophagy are fundamental cell biology processes, establishing the system in different plant species other than *Arabidopsis* will benefit science and society in the long term.

Functional studies of endomembrane system during the developmental program and environmental response have been just started. We hope this research collection can open a door to researchers who are willing to bridge the gap between macroscopic/physiological analysis and subcellular details.

## Author contributions

RL wrote the manuscript. All authors revised and approved the final version.

## Funding

RL was supported by Guangdong Innovation Research Team Fund (2016ZT06S172) and Shenzhen Sci-Tech Fund (KYTDPT20181011104005 and JCYJ20210324105004011). YM was supported by Singapore Ministry of Education (MOE) Tier 1 grant (RT11/20; RG32/20). YF was supported by the National Natural Science Foundation of China (32061143018).

## Conflict of interest

The authors declare that the research was conducted in the absence of any commercial or financial relationships that could be construed as a potential conflict of interest.

## Publisher's note

All claims expressed in this article are solely those of the authors and do not necessarily represent those of their affiliated organizations, or those of the publisher, the editors and the reviewers. Any product that may be evaluated in this article, or claim that may be made by its manufacturer, is not guaranteed or endorsed by the publisher.

